# Identification of *Clostridioides difficile* and *Bacillus cereus* surrogates to assess the sporicidal activity of industrial laundering processes

**DOI:** 10.3389/fbioe.2026.1922833

**Published:** 2026-08-28

**Authors:** Caroline Cayrou, Katie Laird

**Affiliations:** Infectious Disease Research Group, Leicester School of Pharmacy, De Montfort University, Leicester, United Kingdom

**Keywords:** *Bacillus cereus*, *Clostridioides difficile*, industrial laundry, spore, surrogate

## Abstract

Industrial laundry processes are a critical control point for microbiological risk management on reusable textiles, particularly in healthcare and other high-risk settings. Spore-forming bacteria such as *Clostridioides difficile* and *Bacillus cereus* present a challenge to laundering efficacy due to their resistance to thermal and chemical stresses. Validation of sporicidal performance is constrained by the absence of standardized test methods and the biohazard risks associated with using pathogenic spores at industrial scale. This study aimed to identify a safe, technically representative surrogate organism for use in bioindicator-based validation of sporicidal efficacy in industrial laundering processes. Spores of selected candidate organisms (*Metaclostrioides mangenotii*, *Clostridium sporogenes*, and *Bacillus licheniformis*) were produced and purified alongside *C. difficile* and *B. cereus*. Chemical resistance was assessed using in-solution carrier tests and full in-wash bioindicator testing under representative commercial laundry conditions (60 °C, 10 min), using a commercial detergent system and peracetic acid. Minimum inhibitory concentrations (MICs) for peracetic acid were determined to compare intrinsic resistance profiles. All species were inactivated at the in-wash peracetic acid concentration (131 ppm), with substantial differences in resistance observed at lower concentrations; *C. difficile* and *B. cereus* exhibited the highest MICs (131 ppm). *Bacillus licheniformis* demonstrated resistance profiles comparable to the target pathogens during detergent and in-wash testing, while offering a reduced biosafety risk (BSL 1). These findings support *B. licheniformis* as a practical, safer surrogate for sporicidal validation of industrial laundry processes, enabling robust process assurance while reducing operational and biohazard risks during routine testing.

## 1 Introduction

Industrial laundry processes play a critical role in controlling microbial contamination on reusable textiles, particularly within the healthcare and hospitality sectors, where decontamination failure can lead to transmission of infectious disease. Validating the microbiological performance of these processes requires reproducible, scalable tools that accurately reflect in-process conditions while remaining safe for routine industrial use. Bioindicator-based approaches offer a robust means of assessing process efficacy and can effectively evaluate bactericidal performance in commercial laundering systems (Owen et al., 2024). Building on this earlier work describing the development of a standardized bioindicator for healthcare laundry validation, this study addresses the more stringent challenge of sporicidal assurance by developing and assessing an appropriate spore-based bioindicator for industrial laundering processes.

Spores are resistant to various environmental pressures, including desiccation, high temperature, and antiseptics (Setlow, 2014; Setlow and Christie, 2023). Because of their high resistance, they can survive for years in the environment and are difficult to remove with standard cleaning processes, including laundering of contaminated textiles (Tarrant et al., 2018; Tarrant et al., 2022). Several cases of *C. difficile* and *B. cereus* outbreaks have been linked to linen and laundry decontamination failures or cross-contamination. *B. cereus* outbreaks associated with linen have occurred in hospitals in France, the United Kingdom, Italy, Singapore, and Malaysia (Hosein et al., 2013; Bosica et al., 2025; Manin et al., 2026; Balm et al., 2012; Shunmugarajoo et al., 2020). The outbreaks were resolved by changing laundry practices, including more stringent laundry processes and modifying linen storage conditions. In 2013, a C*. difficile* outbreak associated with a laundry machine malfunction occurred in a hospital in the United States (Sooklal et al., 2014).

Currently, in the United Kingdom, no standardized method exists to specifically assess the sporicidal activity of industrial laundry processes. A standardized method based on free contaminated swatches in wash systems is available to evaluate the antimicrobial performance of laundering processes (British Standards Institution, 2022); however, this method does not address bacterial spores. In contrast, a bioindicator-based test method for assessing bactericidal performance under in-use laundry conditions has recently been developed (Owen et al., 2024) and is being included as a referenced test method within BS EN 14065 Risk Analysis and Biocontamination Control (RABC) in Laundries (British Standards Institution, 2016). While this represents an important advance in process control and verification, the absence of a corresponding sporicidal assessment method remains a critical gap in current laundry risk management frameworks. Using open swatches contaminated with pathogenic spore-forming organisms such as *C. difficile* or *B. cereus* to represent laundry-associated microbiological risk presents a significant biohazard in industrial decontamination validation. This risk is especially high when personnel handle free-contaminated swatches before and after processing, posing exposure hazards. Moreover, a laundering process that fails to achieve the required level of microbial reduction risks contaminating the laundering system itself, necessitating extensive decontamination and operational disruption. Encapsulated bioindicators reduce handler exposure during testing; however, breach or damage to the enclosure during washing still presents a risk to both operators and equipment. Using non-pathogenic or low-risk surrogate organisms that closely mimic the temperature and chemical resistance characteristics of clinically relevant spores offers a safer and more practical approach for validating sporicidal performance in industrial laundry processes.

Currently, no surrogates have been used to assess *C. difficile* or *B. cereus* decontamination in the laundering process. Surrogates have been developed for other spore-forming bacteria, such as *Clostridium botulinum* and *Bacillus anthracis*, or for *C. difficile* to test food processing and disinfectant activity (Hu and Gurtler, 2017; Omidbakhsh, 2010; McSharry et al., 2021). More genetically related or non-pathogenic representatives of the *Clostridium* and *Bacillus* genera could also be potential good surrogate candidates for industrial laundry decontamination efficacy testing.

This study evaluates the chemical resistance of selected spore-forming bacteria and benchmarks their resistance profiles against those of *C. difficile* and *B. cereus*, aiming to identify a safe and technically appropriate surrogate organism for validating sporicidal efficacy in industrial laundry processes.

## 2 Methods

### 2.1 Isolate selection

The criteria for the selection of the isolates were: i) genetically closely related to *C. difficile* or *B. cereus*; ii) non-pathogenic organisms, BSL-1 if possible; iii) previously used in an industrial setting for similar or related use.

Four bacterial species were selected: *Metaclostrioides mangenotii*, which is genetically closest to *C. difficile*; *Clostridium sporogenes*, which is not as closely related to *C. difficile* but is already used in the food industry as a surrogate for *Clostridium botulinum*; *Clostridium acetobutylicum*, which is used to produce solvents; *Bacillus licheniformis,* which is used in microbial-based cleaning products.

The isolate codes, biohazard category, and previously known usage of the selected organisms are listed in Table 1.

**TABLE 1:** Bacteria species selected as potential surrogates for *Clostridioides difficile* and *Bacillus cereus*.

Bacteria species	Isolate code	Biohazard category*	Industrial/commercial use
*Metaclostrioides mangenotii*	NCIMB 10639§ ATCC25761 DSM1289 VPI4622	BSL-1 ACDP-2 RG 1	NA
*Clostridium sporogenes*	NCIMB 10696§ ATCC 3584 DSM 795 IFO 13950 JCM 1416 VPI 3046	BSL-1 ACDP-2 RG 2	Surrogate for *C. botulinum* in the food industry (Hu and Gurtler, 2017). Surrogate for disinfectant testing (Omidbakhsh, 2010; McSharry et al., 2021)
*Clostridium acetobutylicum*	NCIMB 13357§ ATCC 824 DSM 792 NRRLB 527 VKMB 1787	BSL-1 ACDP-1 RG 1	Solvent production (Oehlenschläger et al., 2025)
*Bacillus licheniformis*	NCIMB 9375§ ATCC 14580 CCM 2145 CECT 20 CIP 52.71 DSM 13 IAM 13417 IFO 12200 JCM 2505 NCTC 10341 NCDO 1772 WDCM 00068	BSL1 ACDP-2 RG 1 (+)	Same species isolates used in microbial-based cleaning products (Wormald et al., 2023; Syed and Buddolla, 2025) or probiotic supplements for humans and animals (Gweon et al., 2025; Muras et al., 2021)

§ Isolates used in this study.

* BSL, Biosafety level (ATCC-US, classification); ACDP, Advisory Committee on Dangerous Pathogens (NCIMB-UK, classification). RG, Risk group (DSMZ-German classification), (+) = risk of opportunistic infection predominant in severely immunocompromised individuals.

NA, not applicable.

### 2.2 Chemical selection

Six chemical solutions were tested in this study. The chemicals were selected to represent those used by commercial launderers. Sodium dodecyl sulfate (SDS) at 0.05% was selected as it is a common detergent found in laundry products. Peracetic acid at 131 ppm is a disinfectant and a bleaching agent used in laundering processes. In addition, peracetic acid has been previously reported to be effective against spore-forming bacteria (McSharry et al., 2021). A commonly used three-part industrial laundry disinfectant (ILD) (16 mL/kg of textile) which can be used at low temperatures was also selected (proprietary composition). The ILD solution is prepared by mixing three solutions: 5% C1 (unknown), 7.85% C2 (containing hydrogen peroxide), and 9.84% C3 (containing sodium hydroxide in deionized water). Because some components of the solutions forming the IDL chemistry were known (hydrogen peroxide and sodium hydroxide), they were also tested individually.

### 2.3 Bacterial growth

*Clostridium sporogenes, C*. *acetobutyricum*, *M*. *mangenotii*, and *C. difficile* were grown on brain heart infusion agar (BHI) (Oxoid, CM1136B) enriched with 5% defibrinated horse blood (EO Labs/Scientific Laboratory Supplies, MED1302) at 37 °C under anaerobic conditions (10% CO_2_, 5% H_2_, and 85% N_2_). The growth media were reduced in anaerobic conditions 24 h before use.

*Bacillus licheniformis* and *B. cereus* were grown on BHI agar at 37 °C in aerobic conditions.

### 2.4 Spore production and spore purification

Egg-yolk agar (for 1 L: 20 g bacteriological peptone, 5 g NaCl, 5 g tryptone, 5 g yeast extract, 100 mL 50% egg yolk emulsion, and 20 g agar) was pre-reduced in anaerobic conditions, then inoculated with 10 µL of a fresh culture of the isolate. The inoculated media were then incubated at 37 °C for 2 weeks. For *B. licheniformis*, a BHI agar plate was inoculated with 10 µL of a fresh culture and incubated for 2 weeks. After 1 week, the sporulation progression was monitored under a microscope.

After the incubation period, the solid media were washed using 5–10 mL of sterile distilled water, and the wash solution was transferred to a centrifuge tube. Samples were then centrifuged at 3,220 × *g* for 15 min. The supernatant was discarded, and the pellet was resuspended in 5 mL of sterile distilled water and centrifuged again at 3,220 × *g* for 15 min. The washing process was performed four times. After the final wash, the pellets were resuspended in 2 mL of sterile distilled water.

To kill any remaining vegetative cells, the spore suspensions were incubated at 80 °C for 15 min for *C. sporogenes* and *B. licheniform*is and at 65 °C for 15 min for *C. difficile* and *M. mangenotii*.

*Bacillus cereus* sporulation was performed in broth. A single colony of a fresh *B. cereus* culture was transferred to 100 mL of 2XSG broth (For 1 L: 16 g nutrient broth, 2 g KCl, 0.5 g MgSO_4_, 7H_2_O; after autoclaving, add 1 mL each of sterile 1 M Ca(No_3_)_2_, sterile 0.1 M MnCl_2_, and sterile 1 mM Fe SO_4_ and 2 mL of sterile 50% glucose). The inoculated broth was then incubated at 37 °C at 180 rpm for 4 days. After incubation, the culture was centrifuged at 3,000 × *g* for 30 min. The supernatant was discarded, and the pellet was resuspended in 10 mL sterile distilled water. The spore suspension was then centrifuged at 3,000 × *g* for 30 min. The wash process was repeated a total of three times. The remaining vegetative cells were eliminated by heating the purified spore suspension at 70 °C for 30 min, followed by an incubation at 90 °C for 10 min.

A total of three different spore suspensions were produced for each organism. The spore suspensions were then stored at +4 °C until used.

### 2.5 Determination of the sporulation rate

The sporulation rate was determined by sampling the spore suspension before the purification step and counting vegetative cells and spores using a counting chamber under a microscope.

$$Sporulation\;rate=\frac{Total\;count\;spores}{Total\;count\;cells}.$$

### 2.6 Bacteria germination and determination of germination rates

All the anaerobic bacteria spores were germinated using pre-reduced BHI enriched with horse blood and 0.1% taurocholic acid. The aerobic bacteria were germinated using BHI agar.

To determine the germination rate, the spore suspension concentration was calculated using a counting chamber under a microscope. Then, a serial dilution of the spore suspension was performed, and 500 μL of the dilution was spread-plated in duplicate on BHI agar plates for aerobic bacteria and on BHI agar plates enriched with 5% defibrinated horse blood and 0.1% taurocholic acid for the anaerobic bacteria. The plates were incubated at 37 °C for 24 h–48 h under the appropriate atmospheric conditions. After growth, a viable count was performed.

$$Germination\;rate=\frac{Viable\;count}{Total\;count\;spores}.$$

The germination viable count was then used as the spore concentration.

### 2.7 In solution chemical resistance testing

Spore suspensions were calibrated in sterile distilled water to 10^7^ spores/mL.

Sterile 1-cm^2^ cotton swatches were then inoculated with 20 μL of the appropriate spore suspension. The inoculated swatches were then dried for 18 h at room temperature.

After drying, the swatches were transferred to 20 mL glass tubes prewarmed at 60 °C containing 10 mL of either water, ILD (prepared considering 4 L of water/kg of textile), 0.2 mL/l C1, 0.0314 mL/l C2, 0.3936 mL/l C3, 131 ppm peracetic acid, or 0.5% SDS solution. The inoculated swatches were incubated at 60 °C for 10 min. After incubation, the swatches were transferred to 5 mL of neutralizing solution (3% Tween 80, 0.85% sodium chloride, 0.5% sodium thiosulfate, 0.3% lecithin, and 0.1% tryptone), shaken for 30 s, and incubated at room temperature for 5 min. After incubation, the swatch suspension was shaken for 2 seconds, then serially diluted (1:10) in phosphate buffer saline (PBS) to 10^−2^. To determine spore survival, 500 μL of the swatch suspension was spread-plated in duplicate onto the appropriate germination media, and 100 μL of the dilutions was spread-plated in duplicate on the appropriate germination plate. After inoculation, the inoculated plates were incubated in the appropriate conditions for 24 h (*C. difficile, C. sporogenes, B. cereus*, and *B. licheniformis*) and 48 h (*M. mangenotii*). Viable counts were then determined. The plates were incubated for an additional 24 h to confirm the colony morphology or allow any delayed growth to develop. A control untreated swatch was directly transferred to 5 mL neutralizing solution and processed as described above.

### 2.8 In-wash chemical resistance testing

For the in-wash chemical resistance testing, inoculated swatches were prepared as described in Section 2.7, In-solution chemical resistance testing. After drying, the swatches were sealed between two polyethersulfone (PES) membranes to form a bioindicator (Owen et al., 2024). The bioindicators were then placed inside a polycotton pillowcase. The pillowcase was placed in an extractor washer (JLA 16) alongside 4 kg of polycotton makeweights. The bioindicators were washed in a 10-min wash cycle at 60 °C with either water only, ILD (4 kg dose), or peracetic acid (131 ppm).

After washing, the swatches were transferred into 5 mL of PBS and processed as described in Section 2.7, In-solution chemical resistance testingthe “In solution chemical resistance testing” section. An untreated swatch was also processed as previously described as a control.

### 2.9 Minimum inhibitory concentration testing

The peracetic solution was serially diluted 1:2 from 131 ppm to 4.2 ppm. The serially diluted peracetic acid solutions were prewarmed at 60 °C, and spore chemical resistance was tested as described in Section 2.7, In-solution chemical resistance testing section, with the exception that only the neat swatch suspension was plated. Water was used as a control. After incubation, only the presence of growth was considered, and no viable counts were performed.

### 2.10 Statistical analysis

The normality of the data was assessed using both a Q-Q plot and a Shapiro test. The spore reduction observed for the surrogate candidates was compared against *B. cereus* and *C. difficile* using an independent samples Kruskal–Wallis test, with a Benjamin–Hochberg correction for multiple comparisons and a one-way ANOVA test with a Dunnett’s *post-hoc* test. All statistical tests were done using SPSS (version 31, IBM) with statistical significance established at *p*-value ≤0.05. Five species were explored with three biological replicates per test for each.

## 3 Results

### 3.1 Bacteria growth, sporulation, and germination parameters

All anaerobic bacteria grew on BHI agar supplemented with 5% horse blood in 24 h. *Clostridium acetobutylicum* growth was inconsistent. For this reason, *C. acetobutylicum* was excluded from further testing.

The sporulation rate obtained during the experiment was on average 80%, 94%, 83%, 99.6%, and 97% for *C. difficile*, *M. mangenotii*, *C. sporogenes*, *B. cereus*, and *B. licheniformis*, respectively.

The germination rates observed were 0.05% for *C. difficile*, 0.02% for *M. mangenotii*, 48% for *C. sporogenes*, 99.22% for *B. cereus*, and 18% for *B. licheniformis*.

### 3.2 In solution chemical resistance

**FIGURE 1:**
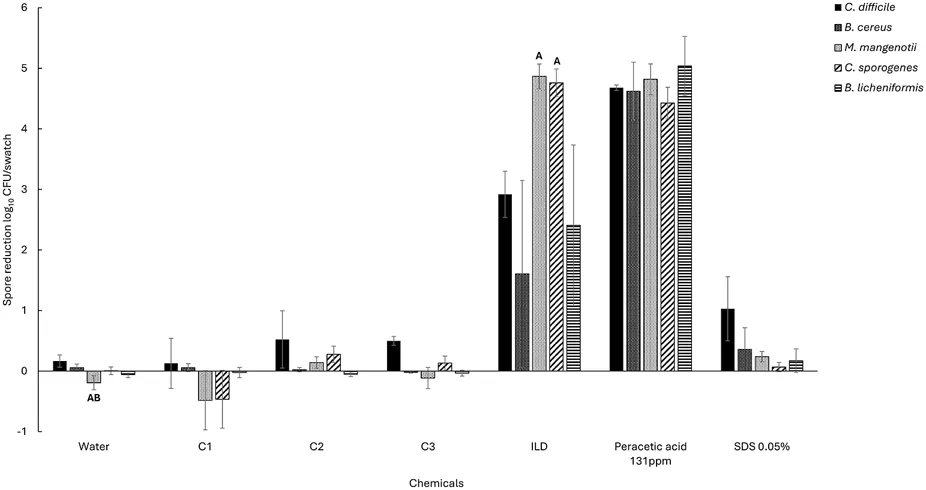
In-solution chemical resistance. A = Significantly different (*p* ≤ 0.05) compared to *Bacillus cereus* and B = Significantly different (*p* ≤ 0.05) compared to *Clostridioides difficile*. The arrow bars represent the standard deviation (n = 3, ±SD).

Three chemicals were tested for their sporicidal activity: ILD, 0.5% SDS, and peracetic acid. Due to the ILD being formed of three different chemistries, C1 (unknown composition), C2 (hydrogen peroxide), and C3 (sodium hydroxide), the solutions were also tested independently to assess their individual sporicidal activity. Water was used as a control to ensure incubation at 60 °C did not affect spore survival. *Clostridioides difficile*, *B. cereus*, and *B. licheniformis* exhibited higher resistance to ILD (1.6–2.9 log_10_ reduction) than *M. mangenotii* and *C. sporogenes* (>4 log_10_ reduction) (Figure 1). However, all the isolate spores were eliminated when incubated in peracetic acid (Figure 1) (<limit of detection = 5 CFU/swatch). When the swatches were incubated in water, C1, C2, or C3, a spore reduction of < 1 log_10_ was observed (Figure 1) for all species.

### 3.3 In-wash chemical resistance

In order to confirm the results observed in the in-solution chemical resistance investigation and to assess the bioindicator method, bioindicators containing the isolates were washed using ILD or peracetic acid during an extractor wash process.

**FIGURE 2:**
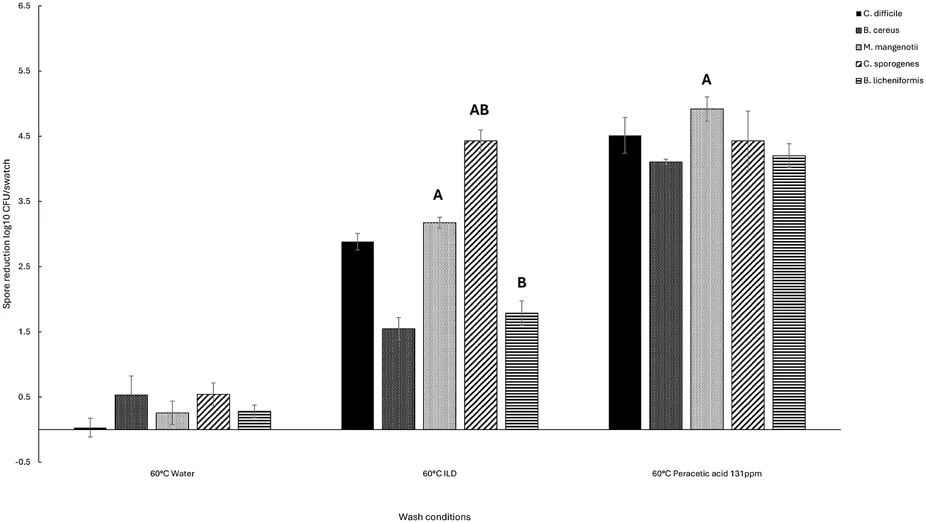
In-wash chemical resistance. A = Significantly different (*p* ≤ 0.05) compared to *Bacillus cereus* and B = Significantly different (*p* ≤ 0.05) compared to *Clostridioides difficile*. The arrow bars represent the standard deviation (n = 3, ±SD).

As observed in the in-solution test, all isolates were eliminated during the peracetic acid wash. *Clostridium sporogenes* was fully eliminated when using ILD (Figure 2). As previously observed, *C. difficile*, *B. cereus*, and *B. licheniformis* showed resistance to the ILD (<3 log_10_ reduction). *Metaclostrioides mangenotii* showed higher in-wash than in-solution resistance, with a bacterial reduction of slightly higher than 3 log_10_ (Figure 2).

### 3.4 Minimum inhibitory concentration

Peracetic acid exhibited a total inhibition of the spores during the in-solution and in-wash tests. In order to compare the different species’ resistance to peracetic acid solution, the peracetic MIC was determined for each species. *Bacillus cereus* and *C. difficile* exhibited the highest resistance to peracetic acid, as only the full-strength concentration (131 ppm) was able to eliminate the spores (Table 2). *Bacillus licheniformis* exhibited sensitivity to half the concentration of peracetic acid (65.5 ppm), as did *M. mangenotii,* but 1/4 of the in-wash concentration (32.8 ppm) lost its efficacy against both isolates. *Clostridium sporogenes* was the most susceptible to peracetic acid, as the in -wash concentration needed to be reduced by 1/16 (8.2 ppm) before consistent growth appeared (Table 2).

**TABLE 2:** Minimum inhibitory concentration testing of peracetic acid (n = 3).

Peracetic acid dilution (concentration)	*C. difficile*	*B. cereus*	*M mangenotii*	*C. sporogenes*	*B. licheniformis*
Neat* (131 ppm)	---	--+	---	---	---
1/2 (65.5 ppm)	+++	+++	---	-	--+
1/4 (32.8 ppm)	+++	+++	+++	-	+++
1/8 (16.4 ppm)	​	​	+++	--+	​
1/16 (8.2 ppm)	​	​	​	-++	​
1/32 (4.1 ppm)	​	​	​	+++	​

* Neat, 0.374 mL/L of 35% peracetic acid solution.

-, No growth.

+, Growth.

## 4 Discussion

All spores tested were resistant to temperature and some of the chemicals used in industrial laundering processes. Thus, no bacterial reduction was observed when the spores were treated at 60 °C for 10 min in water, the individual solutions of the ILD, or with SDS.

The ILD showed variable results, exhibiting higher sporicidal activity against anaerobic spore-forming bacteria (2.9–4.3 log_10_ CFU/swatch reduction) than against aerobic spore-forming bacteria (1.5–1.8 log_10_ CFU/swatch reduction). The composition of ILD is proprietary, making it difficult to identify the mechanism behind this partial sporicidal activity. However, it might be due to differences in the spore structure or metabolism that make the anaerobic spores more susceptible to the ILD. Only a few studies directly compared resistance to chemical and physical pressure between aerobic and anaerobic spores. They showed that the resistance levels to thermal treatment, acid hydrolysis, or alkaline hydrolysis were species-dependent and could not be associated with anaerobe and aerobe groups (Ahn et al., 2007; Heckler et al., 2026).

All the spore-forming bacteria species tested were sensitive to peracetic acid at the in-wash concentration (131 ppm). Greater variation could be observed in their MIC concentrations. *Clostridium sporogenes* showed the highest susceptibility with an MIC at 16.4 ppm, followed by *M. mangenotii* and *B. licheniformis* (65.5 ppm). *Bacillus cereus* and *C. difficile* exhibited the highest resistance (131 ppm). Although all species were inactivated at the in-wash peracetic acid concentration, the differences in MICs highlight variation in intrinsic spore resistance. This distinction is critical for process validation, as a suitable surrogate must reflect the upper range of resistance exhibited by clinically relevant spores such as *C. difficile* and *B. cereus*, ensuring that laundering processes validated using the surrogate provide a conservative and reliable measure of sporicidal efficacy.

*Bacillus cereus* exhibited the highest resistance to the chemical tested (MIC of 131 ppm for peracetic acid and only 1.55 log_10_ reduction by ILD in wash) among all the spore-forming bacteria species (Figure 2; Table 2). This high resistance could be one of the reasons explaining why there are frequent cases of hospital *B. cereus* outbreaks associated with linen contamination, which were resolved by more stringent infection control practices of the laundry processes (Barrie et al., 1994; Balm et al., 2012; Bosica et al., 2025; Hosein et al., 2013). *Metaclostrioides mangenotii* and *C. sporogenes* exhibited significantly lower resistance to the chemicals tested (in-wash ILD 3.2 and 4.4 log_10_ reduction, respectively, and peracetic acid MIC at 65.5 ppm and 8.2 ppm, respectively) (Figure 2; Table 2). *Clostridium sporogenes* has been previously reported to be less resistant to peracetic acid than *C. difficile* (McSharry et al., 2021). The significantly lower resistance exhibited by *M. mangenotii* and *C. sporogenes* to standard laundry disinfectant and peracetic acid in the wash means that they cannot be used as surrogates for *B. cereus* and *C. difficile* (Figure 2; Table 2).

Overall, this study indicates that *B. licheniformis* was the most appropriate surrogate to be used in bioindicators for industrial laundry decontamination validation. *Bacillus licheniformis* is classified as a biosafety level 1 (BSL-1) organism in multiple jurisdictions and is not considered a primary human pathogen (Deniz et al., 2025), despite infrequent reports of opportunistic contamination or infection in clinical settings. Importantly, the species has an established history of safe use in industrial, probiotic and microbial-based cleaning applications (Wormald et al., 2023; Syed and Buddholla, 2025; Muras et al., 2021; Gweon et al., 2025), supporting its suitability for routine handling and large-scale process testing. Compared with *C*. *difficile* and *B*. *cereus,* recognized human pathogens, *B. licheniformis* represents a substantially reduced biohazard risk for personnel and laundering systems during sporicidal validation testing. Although *B. licheniformis* exhibited slightly lower resistance to peracetic acid (65.5 ppm) than *C. difficile* and *B. cereus* (both 131 ppm), its overall resistance profile, combined with its favorable safety characteristics, makes it the most practical and appropriate surrogate for evaluating sporicidal efficacy in industrial laundry processes (Figure 2; Table 2).

A method based on bioindicators containing four cotton swatches each inoculated with known concentrations of the bacteria (10^6^ log_10_ CFU to 10^3^ log_10_ CFU) has been developed to test the bactericidal activity of the laundering processes (Owen et al., 2024). This method allows for a semi-quantitative estimation of the bactericidal activity by checking each swatch for growth after the laundering process. The BS EN 14065:2016 Risk Analysis and Biocontamination Control (RABC) in Laundries standard is currently being revised to integrate this bioindicator method for testing the bactericidal activity and sporicidal activity of the laundering processes. Identifying a surrogate for *B. cereus* and *C. difficile* is essential for developing the bioindicator method to test the sporicidal activity of the laundry processes.

A limited set of chemicals was tested against *C. difficile*, *B. cereus*, and *B. licheniformis*, so it is only possible to confirm that *B. licheniformis* can be used as a surrogate when using those industrial laundry chemicals. If another disinfectant were to be used with industrial laundering processes, additional tests should be performed to assess the resistance profile and confirm that *B. licheniformis* remains an appropriate surrogate.

This study identifies for the first time *B. licheniformis* as a potential surrogate for *C. difficile* and *B. cereus* to be used in laundry bioindicators to assess the sporicidal activity of laundry processes.

## Data Availability

The original contributions presented in the study are included in the article/supplementary material, further inquiries can be directed to the corresponding author.
